# Anatomic versus reverse total shoulder replacement with an intact rotator cuff: a comparative musculoskeletal simulation study of glenohumeral joint biomechanics

**DOI:** 10.1186/s42836-026-00432-4

**Published:** 2026-10-01

**Authors:** Iman Soodmand, Thomas Tischer, Sven Herrmann, Tilman Klose, Leo Ruehrmund, Christoph Lutter, Christoph Woernle, Rainer Bader, Maeruan Kebbach

**Affiliations:** 1 Research Laboratory for Biomechanics and Implant Technology, Department of Orthopaedics, Rostock University Medical Center, 18057 Rostock, Germany; 2 Department of Orthopaedics and Trauma Surgery, Malteser Waldkrankenhaus, 91054 Erlangen, Germany; 3 Institute for Biomechanics, BG Unfallklinik Murnau, 82418 Murnau, Germany; 4 Chair of Technical Dynamics, University of Rostock, 18059 Rostock, Germany; 5 Department Life, Light & Matter (LL&M), University of Rostock, 18059 Rostock, Germany

**Keywords:** Anatomic total shoulder arthroplasty, Reverse total shoulder arthroplasty, Computational simulation, Contact mechanics, Glenohumeral joint biomechanics

## Abstract

**Background:**

Reverse total shoulder replacement (rTSR) is increasingly being conducted, including in selected elderly patients with intact-cuff osteoarthritis, due to concerns about subsequent cuff failure or glenoid component loosening after anatomic total shoulder replacement (aTSR). However, the biomechanical differences between rTSR and aTSR remain insufficiently understood. This computational study aimed to compare the biomechanics of aTSR and rTSR with an intact rotator cuff under identical conditions, representing selected intact-cuff osteoarthritis cases in which either implant design may be considered.

**Methods:**

A validated musculoskeletal multibody simulation framework of the upper extremity was used to compare the glenohumeral (GH) biomechanics during arm elevation in the scapular plane. The GH joint was modeled in detail with six degrees of freedom and explicit contact between the articulating implant components. This enabled the evaluation of muscle forces, deltoid-to-cuff force ratio, GH joint contact forces, shear-to-compression joint force balance, contact area, contact pressure, center of pressure, and GH translations.

**Results:**

Compared with aTSR, rTSR reduced the mean middle deltoid muscle force by 16% and decreased overall rotator cuff force requirements, while increasing deltoid-to-cuff dominance throughout most of the motion. Furthermore, rTSR showed 26.5% and 33.4% lower compressive and resultant GH contact forces, but earlier and 98.7% higher peak superior shear force and a higher shear-to-compression joint force ratio. Contact pressure was 3.5 times lower in rTSR, whereas the contact area was nearly three times larger. Center-of-pressure trajectories differed substantially between designs, in both magnitude and direction. GH translations were more constrained in rTSR, with negligible posterior-anterior displacements and smaller overall translations.

**Conclusion:**

Under an intact-cuff condition, aTSR and rTSR produced altered mechanical strategies for arm elevation. rTSR was characterized by more deltoid-dominant loading, lower joint compression, altered shear force patterns, and more constrained joint translations. This study provides a biomechanical insight for interpreting clinically relevant differences between the two mechanically distinct TSR designs. These findings may help surgeons better understand design-specific trade-offs in joint loading, shear-to-compression joint force balance, and muscle function, and may support clinical decision-making, particularly in intact-cuff conditions.

## Introduction

Anatomic (aTSR) and reverse (rTSR) total shoulder replacements are established treatments to relieve pain and improve function in degenerative conditions of the glenohumeral (GH) joint. Over the past decade, international joint registry data [1] have consistently reported rising total shoulder replacement (TSR) volumes, with the rapid growth in the rTSR, which has nearly doubled internationally [1], now representing a larger and growing share of surgeries compared to aTSR.

Comparative studies highlight clear advantages for each design [2–4]. Anatomic designs are typically selected for advanced GH osteoarthritis when the rotator cuff is intact and functional [2]. This design is often preferred in younger patients, including those under 55 years, because it aims to preserve the rotational range of motion [5, 6]. Reverse design, on the other hand, converts the proximal humerus into a socket and the glenoid into a ball by reversing the ball and socket position and was initially developed for cuff tear arthropathy and other deficient-cuff conditions. The indications, however, have expanded to complex proximal humerus fractures, revision surgery, and selected primary osteoarthritis cases [2].

Although rTSR was initially developed for cuff-deficient conditions, its application has expanded to selected older patients with intact-cuff primary GH osteoarthritis, including cases in which concerns exist regarding future rotator cuff failure or glenoid bone loss [7, 8]. Preservation of a functional rotator cuff after rTSR has also been reported in selected intact-cuff osteoarthritis cases [8, 9]. The present study was therefore designed specifically to investigate this intact-cuff clinical condition, for which the biomechanical consequences of choosing rTSR rather than aTSR remain incompletely understood. In this context, contemporary registry analyses indicate that revision outcomes of rTSR and aTSR in intact-cuff osteoarthritis can be broadly comparable in older patients [10].

The clinical follow-up [4], registry-based studies [10, 11], reviews, and meta-analyses [1, 12] have previously characterized outcomes after aTSR and rTSR. Still, a biomechanical comparison remains crucial because the two designs rely on different stabilizing strategies and joint mechanics. Anatomic design aims to restore native anatomy and relies more on rotator cuff force couples to maintain joint stability [2]. In contrast, rTSR commonly medializes and inferiorizes the center of rotation and increases deltoid leverage through a semi-constrained articulation [2]. Previous studies have demonstrated that these differences alter GH joint loading and mechanics [13–22], motivating a comparative study to comprehensively quantify the biomechanical differences between these established TSR designs.

In this regard, direct in vivo measurement of internal GH muscle forces and articular contact mechanics remains technically challenging [23]. Also, experiments on human specimens generally enable the assessment of a restricted set of biomechanical variables [24, 25]. As a result, biomechanical evidence increasingly relies on non-invasive computational modeling [13, 14, 16, 20, 21, 26–28], an efficient research tool for quantifying muscle and joint loading under controlled variables and for comparing implant designs under reproducible conditions. A previous experiment on human specimens combined with patient-specific simulations showed that implant lateralization in rTSR alters muscle lines of action and joint loading compared with aTSR [13]. Other simulations quantified the influence of rotator cuff integrity, finding that a functional rotator cuff can increase compressive joint reaction forces by more than two-times compared with a massive tear [16, 20, 21]. Earlier simulation studies estimated volumetric polyethylene wear [15, 17, 22], and reported up to five times higher wear rates in rTSR than in aTSR [15].

These prior computational studies have provided important insights into the varied biomechanics of aTSR and rTSR. However, many musculoskeletal models represent the prosthetic GH joint as a simplified ball-and-socket articulation, neglecting translations and fixing the joint center of rotation (COR) without explicitly representing the real contact pair geometries and friction between them [13, 19–21, 28, 29]. The latter choice removes the contact constraints that govern load transmission across the contact interface between the implant components and complicates an accurate evaluation of joint contact mechanisms [30]. Also, neglecting translations prevents the evaluation of complex mechanisms of GH joint subluxation [14, 30]. Together, these simplifications limit the ability to quantify GH translations, dynamic joint centering, edge loading, or eccentric contact patterns [29–31]. Therefore, the detailed biomechanical differences between aTSR and rTSR have remained insufficiently understood to date.

The objective of the present study was therefore to comprehensively compare and quantify biomechanical differences between aTSR and rTSR during scapular plane elevation under identical intact-cuff conditions. In this regard, we employed a previously validated musculoskeletal multibody simulation framework of the upper extremity, which represents a GH joint after TSR with explicit articular contact and all rotations and translations [32]. For each design, we estimated internal muscle loading around the GH joint, the deltoid-to-cuff force ratio, contact forces transmitted between the GH implant components, the corresponding instability ratio, contact area, pressure, and center of pressure (COP), and GH joint translations.

## Materials and methods

A previously validated three-dimensional musculoskeletal multibody simulation framework of the upper extremity, the Rostock Shoulder Software-in-Loop (RoSSiL) framework, developed in the multibody simulation environment SIMPACK (v. 2022x, Dassault Systèmes, Vélizy-Villacoublay, France), was used in the present study to compare aTSR and rTSR biomechanics. Implementation details of the framework were provided by Soodmand et al. [32].

**Fig. 1:**
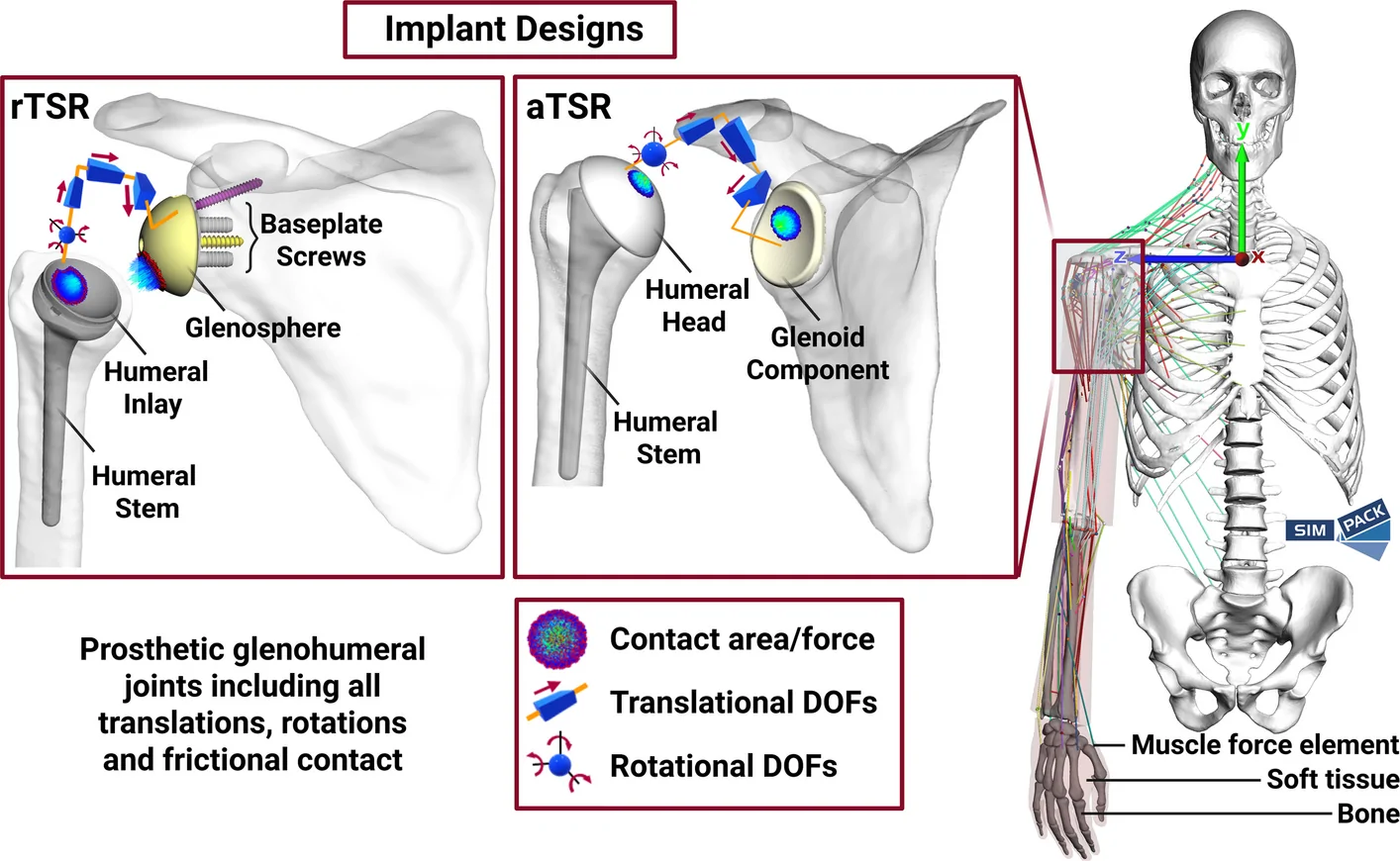
Musculoskeletal multibody model of the prosthetic right upper extremity after anatomic (aTSR) and reverse (rTSR) total shoulder replacements in the software SIMPACK. The right panel shows the right upper extremity musculoskeletal multibody model with 152 discretized muscle force elements (shown as multiple colorful straight strands), seven rigid bones in white, and simplified surrounding soft tissue in transparent dark red. The middle and left panels illustrate the prosthetic GH joint with all rotational and translational degrees of freedom (DOFs) and frictional contact between implant components, for aTSR and rTSR, respectively, enabling detailed investigation of the contact mechanics

Briefly, the skeletal system was modeled as a kinematic chain with seven rigid bone segments, reconstructed from computed tomography data from the Visible Human Male dataset (Fig. 1) [33]. This includes the thorax, clavicle, scapula, humerus, ulna, radius, and hand, with 16 degrees of freedom (DOF). The thorax was considered to be fixed in space, and sternoclavicular and acromioclavicular joints were modeled as ball-and-socket joints. The elbow joint was represented by flexion–extension and pronation-supination, and the wrist joint was modeled as a universal joint. Scapular motion within the shoulder complex was prescribed in the scapular plane in rhythm with clavicular and arm rotations. To model a detailed GH joint, all joint translations and rotations were considered.

The mass and inertia of bones were computed from reconstructed geometries and density estimates for a male subject weighing 58 kg. The surrounding soft tissue was represented by simplified geometric abstractions that contributed to the mass matrix, enabling a more accurate estimation of arm weight (Fig. 1) [32, 34, 35]. The mass and inertia of the implant components were computed from their geometry and material density, and component masses were also included in an accurate estimation of the arm weight.

For aTSR, a commercially available anatomic implant system (Affinis, Mathys AG, Bettlach, Switzerland) with a nonconforming implant design, including a glenoid component size 3 made from Vitamin-E Highly Cross-Linked Polyethylene (VEPE^®^) [36] and humeral head size 52 made from Cobalt Chromium Molybdenum (CoCrMo) [37], was virtually implanted into the scapular and humeral bone stocks following the surgical technique of the manufacturer [37]. Component sizing and placement were verified by shoulder surgeons.

To conduct the comparative biomechanical analyses, the previously developed RoSSiL simulation framework [32] was extended in the present study to include a reverse implant system (Affinis Inverse, Mathys AG, Bettlach, Switzerland) with a glenosphere size 39 made of VEPE^®^ and an Inlay size 39 made of Alumina-Toughened Zirconia (Ceramys^®^) [38]. Implant-specific component designs for aTSR and rTSR are shown in Fig. 1, and material specifications are listed in Table 1.

**Table 1:** Material specifications for contact pairs of the aTSR (Affinis) and rTSR (Affinis Inverse) implant systems

Implant components	Material	E-Modulus [MPa]	ν	ρ [kg·m^−3^]
Humeral Head	CoCrMo	210,000	0.30	8500
aTSR and rTSR Humeral Stems, rTSR Baseplate, and Screws	Ti6Al4V	110,000	0.34	4500
aTSR Glenoid Component, rTSR Glenosphere	VEPE^®^	940	0.46	936
rTSR Inlay	Ceramys^®^	261,000	0.27	5510
	**aTSR**		**rTSR**	
Friction Coefficients	0.07		0.05	

Ceramys^®^, Dispersion Ceramic, Alumina-Toughened Zirconia (ATZ); CoCrMo, Cobalt Chromium Molybdenum; E, Elastic modulus; Ti6Al4V, Titanium-6% Aluminum-4% Vanadium; VEPE^®^, Vitamin-E Highly Cross-Linked Polyethylene; ν, Poisson’s Ratio; ρ, Density

**Fig. 2:**
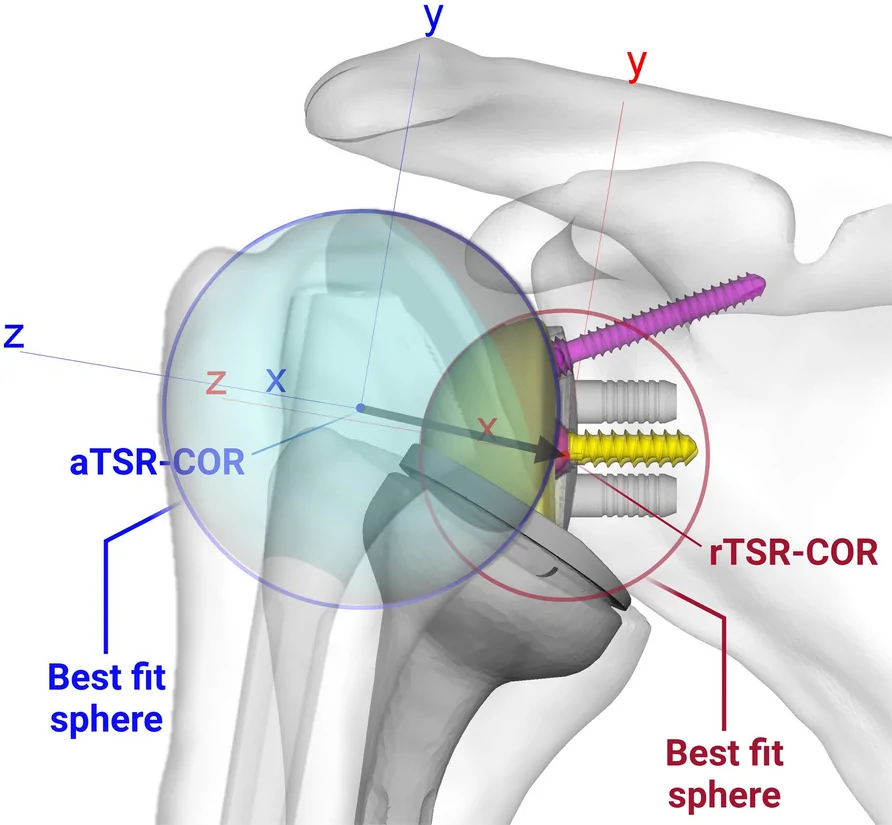
Relocation of the center of rotation (COR) between the anatomic (aTSR) and reverse (rTSR) total shoulder replacement. Schematic of the glenohumeral implant configurations showing the COR for aTSR (blue, left) and rTSR (red, right). The thick black arrow indicates the displacement vector from the aTSR-COR to the rTSR-COR, illustrating medialization and inferiorization of the COR in the rTSR

The center of rotation (COR) for aTSR was defined as the center of the prosthetic humeral head, whereas the COR for rTSR was defined as the center of the glenosphere (Fig. 2). The relocation of COR between designs was quantified as a vector between these two centers.

The musculoskeletal model for both aTSR and rTSR included 48 muscles, discretized into 152 muscle force elements to represent the three-dimensional structure of muscles and the large attachment areas [39]. Muscle origins and insertions were defined according to standard anatomic references [40], mapped to distributed attachment areas, and verified by shoulder surgeons. Muscle paths were guided using via-points and analytical wrapping surfaces.

Muscles were represented by simplified unidimensional Hill-type force elements. Muscle activations were estimated using static optimization that minimized an energy-efficient quadratic cost function, subject to physiological activation bounds, to reproduce the required joint moments for the desired arm elevation in the scapular plane. No rotator cuff muscle deficiency or resection was modeled. A fully functional rotator cuff was retained in both designs for two reasons. First, to represent selected patients with intact-cuff primary GH osteoarthritis in whom either aTSR or rTSR may be considered [7, 8]. Second, to keep the cuff function identical for both designs and thereby investigating the isolated effects of implant design on the GH joint biomechanics. Consequently, the modeled rTSR condition does not represent cuff-tear arthropathy, massive rotator cuff tears, or other cuff-deficient rTSR indications.

In the forward dynamics, the GH articulation was modeled using a polygonal multi-point surface contact model between the implant components, preventing interpenetration of implant components and reproducing roll-slip frictional behavior [41].

The studied arm elevation in the scapular plane was simulated as a 120° thoracohumeral elevation. Sternoclavicular rotation and acromioclavicular orientation were prescribed to align the scapula with the scapular plane. The translational degrees of freedom of the GH joint were not prescribed or directly controlled. Instead, they resulted from the system dynamics, as determined by skeletal inertia, muscle forces, implant geometry, and frictional contact between the articulating components. The GH joint translation was defined as the time-dependent displacement of the centers of best-fitting spheres on the humeral head and glenoid component for aTSR, and on the humeral inlay and glenosphere for rTSR.

To better analyze the relation between the deltoid and rotator cuff muscle groups in each implant design, we defined the Deltoid-to-Cuff ratio (DCR). The DCR further highlights the shift in force dominance between the deltoid and rotator cuff muscles in aTSR and rTSR and enables the evaluation of coordination of muscles spanning the GH joint. It is defined as the sum of forces exerted by the deltoid muscle heads divided by the total forces generated by rotator cuff muscles, with a 1:1 balanced ratio indicating equal total deltoid and cuff muscle forces.

To compare the balance between shear and compressive GH joint loading across anatomic and reverse designs, the instability ratio, a quantitative biomechanical indicator for evaluating the concavity-compression effect [42], was defined as the ratio of shear joint contact force to compression joint contact force, all expressed in the glenoid or glenosphere coordinate systems. For the directional ratios, the posterior-anterior and superior-inferior shear force components, considering the signs, were divided by the magnitude of the compressive force. Thus, the sign indicates the direction of the corresponding shear force component according to the defined coordinate system. The total instability ratio was calculated as the resultant shear force magnitude, i.e., the square root of the sum of squares of the shear force components, divided by the compressive force magnitude, and was therefore always non-negative. A decrease in the ratio indicates more compression-dominant loading, which can contribute to greater joint stability, while an increase in the ratio indicates a greater shear component relative to compression, which suggests a greater potential for joint instability. However, because this force-based biomechanical metric does not account for factors such as implant conformity, geometric constraint, and soft-tissue restraint, it is not interpreted as a direct measure of overall joint stability or clinical dislocation risk in the present study.

## Results

The employed RoSSiL simulation framework revealed the effect of the implant design on the GH joint biomechanics, with a change from aTSR to rTSR resulting in considerable alterations in muscle forces, joint loading, contact mechanics, and joint kinematics. The COR was medialized approximately 27 mm for rTSR compared with aTSR (Fig. 2) [43].

### Muscle forces

**Fig. 3:**
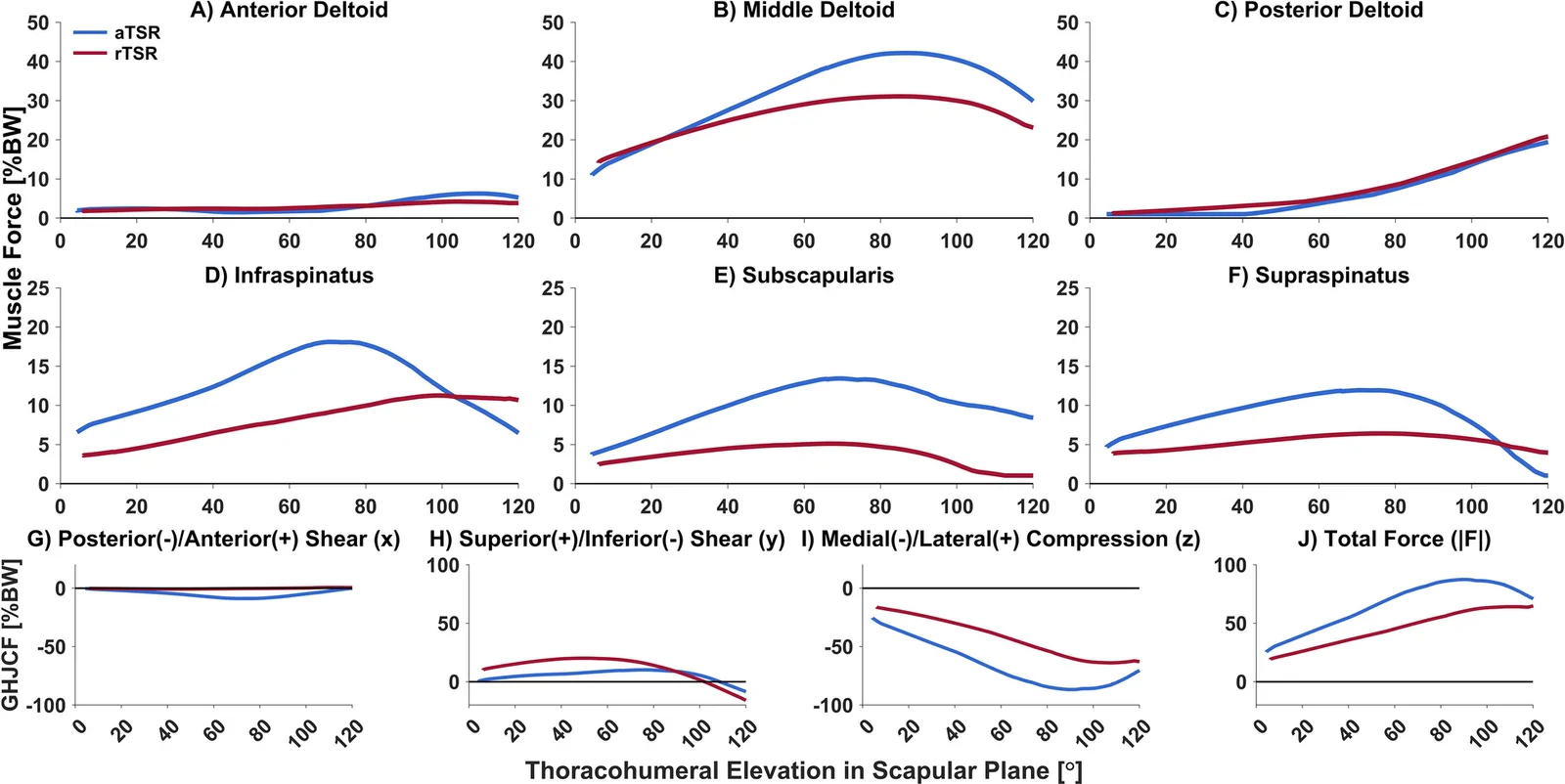
Muscle forces and glenohumeral joint contact forces (GHJCF) during arm elevation in the scapular plane for anatomic (aTSR) and reverse total shoulder replacement (rTSR). The results calculated by the RoSSiL simulation framework in the present study are compared for the aTSR (blue) and the rTSR (red). The top and middle panels present individual muscle forces: **A** anterior deltoid force, **B** middle deltoid force, **C** posterior deltoid force, **D** infraspinatus force, **E** subscapularis force, and **F** supraspinatus force. The bottom panel shows GHJCF components expressed in the glenoid and glenosphere coordinate systems: **G** posterior-anterior shear (x), **H** superior-inferior shear (y), **I** medial–lateral compression (z), and **J** total resultant force magnitude (‖F‖)

Changing from anatomic to reverse design caused no considerable effect on the forces produced by the anterior and posterior deltoids (Fig. 3A, C). Anterior deltoid force remained low through the entire range, below seven% body weight (BW) (Fig. 3A). Posterior deltoid force increased progressively in both conditions (Fig. 3C), reaching a maximum around 20.55%BW. However, less force was required in the middle deltoid in rTSR for arm elevation in the scapular plane (Fig. 3B). The peak force produced by the middle deltoid muscle decreased from 42.16%BW (240 N) at 86° in the aTSR to 31.09%BW (177 N) at 85° in rTSR, and the mean value decreased by 16%. No considerable time shift in the muscle activation patterns of the deltoid muscles was observed.

All predicted rotator cuff muscle force magnitudes were consistently lower in rTSR than aTSR. For the infraspinatus, subscapularis, and supraspinatus muscles, the maximum generated muscle forces were 18.10, 13.43, and 11.93%BW in the aTSR designs and 11.25, 5.11, and 6.41%BW in the rTSR design, respectively. Although infraspinatus and supraspinatus showed a peak around 70° in aTSR, they continued to increase or maintain activation in rTSR as arm elevation progressed, even beyond 70° (Fig. 3D, E).

**Fig. 4:**
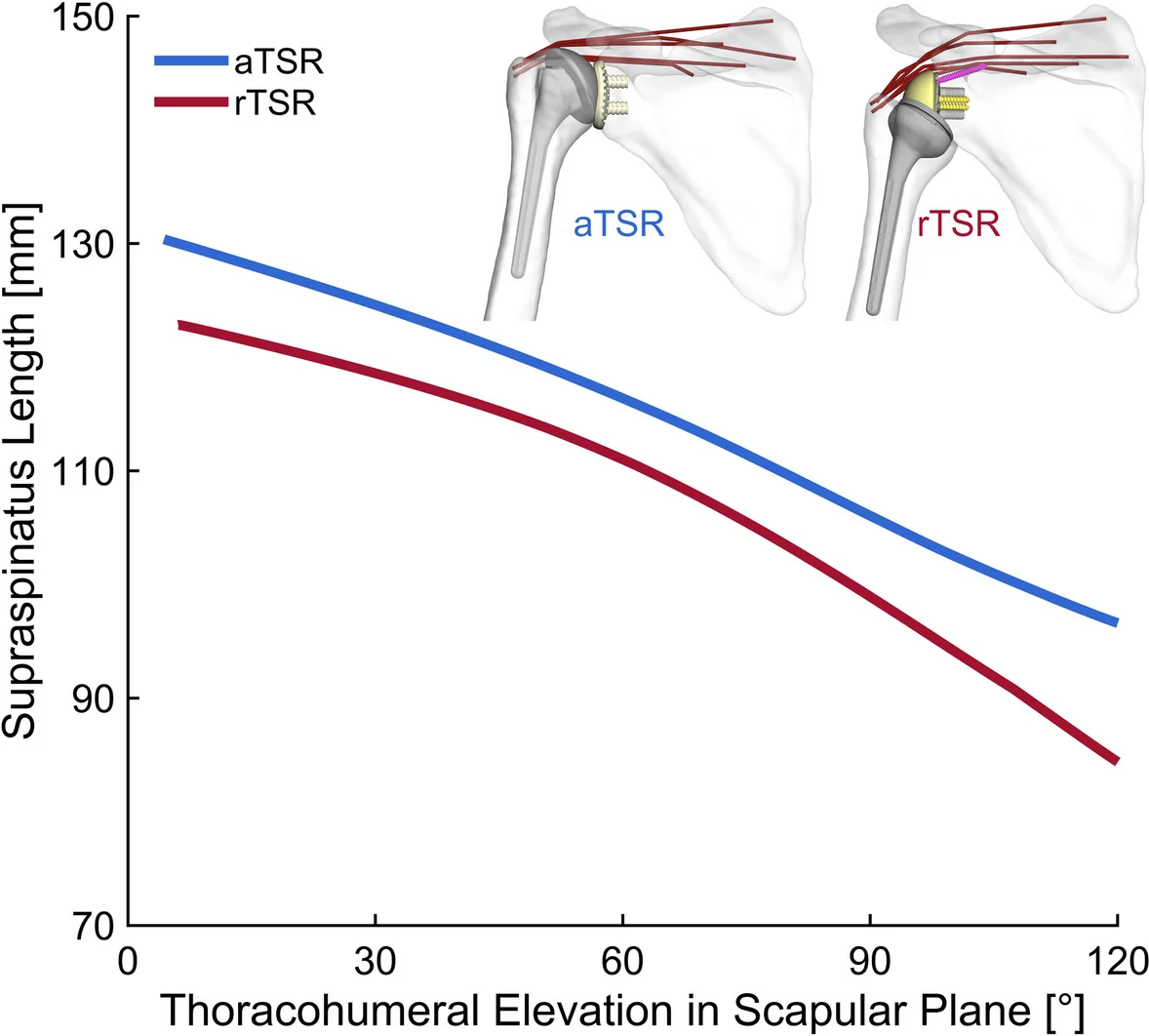
Comparison of the path and length of the supraspinatus muscle after anatomic (aTSR) and reverse total shoulder replacement (rTSR). The results calculated by the RoSSiL simulation framework in the present study are compared for the aTSR (blue) and the rTSR (red). The supraspinatus muscle path is illustrated in the top-right corner for aTSR (left) and rTSR (right)

Implant design and the shifted COR not only changed the muscle forces and directions but also the muscle paths. Among all, the mean supraspinatus muscle length decreased progressively with elevation in both designs (Fig. 4), but remained consistently lower in rTSR than in aTSR across the full motion range, with a 5.78% decrease from aTSR to rTSR at the beginning of motion.

### Deltoid-to-cuff ratio (DCR)

**Fig. 5:**
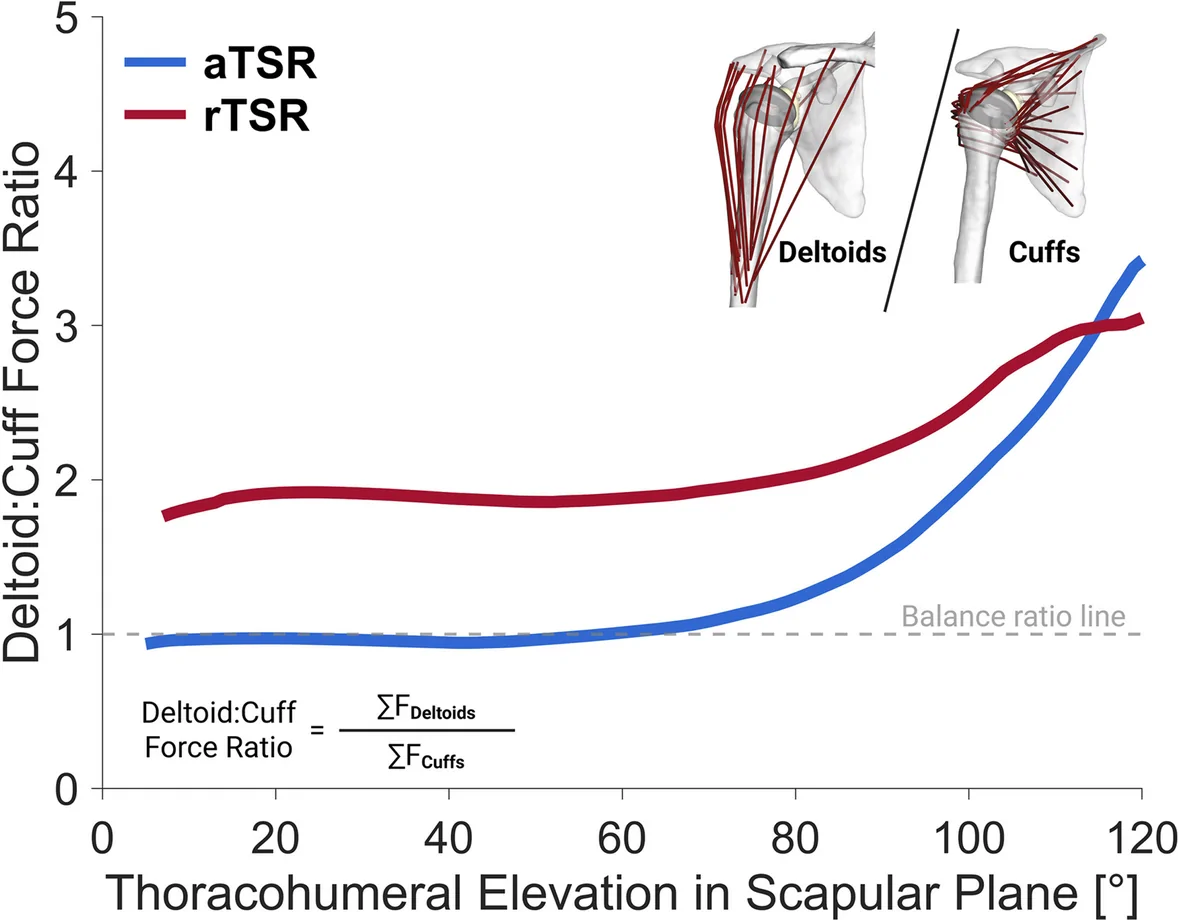
Deltoid-to-Cuff ratio (DCR) during arm elevation in the scapular plane for anatomic (aTSR) and reverse total shoulder replacement (rTSR). The figure compares the DCR, the ratio of the summed deltoid muscle forces to the summed rotator cuff muscle forces (∑F_Deltoids_/∑F_Cuffs_), calculated by the RoSSiL simulation framework. It illustrates the dependence of this ratio on arm elevation for each design. The horizontal, dashed, grey line shows the 1:1 DCR, where the deltoid and rotator cuffs generate equal, balanced forces. In the top right corner, the deltoids and cuff muscles in the musculoskeletal model of the present study are shown. In the bottom-left corner, the mathematical definition of DCR is shown

In the aTSR design, at low elevation angles, the ratio was around 0.95 (Fig. 5), where the cuff was loaded slightly more than the deltoid. The ratio stayed close to the balance line up to a crossover angle of around 58°, after which the deltoid muscle dominated the cuff, and the ratio grew rapidly to the end of the motion range, with 57% deltoid dominance throughout the motion.

In the rTSR design, however, the ratio remained consistently above one throughout the motion. The ratio increased steadily, starting from 1.76, reaching 1.88 at 60°, 2.20 at 90°, and peaking at 3.04 at 120°.

rTSR showed a higher ratio at low elevation, remaining around 1.8–2.0 up to 70°, whereas aTSR stayed close to the balance line with 0.9–1.1 over the same range. Beyond 70°, the ratio increased in both designs, but the rise was sharper in aTSR.

### Glenohumeral joint contact forces

Posterior-anterior shear glenohumeral joint contact force (GHJCF) (Fig. 3G) remained close to zero throughout elevation, with a maximum of 8.87%BW at 70° for aTSR and almost zero values for rTSR. Superior shear force (Fig. 3H) in rTSR increased earlier, peaking at 20.09%BW at 50°, 98.7% larger than aTSR, which increased more gradually and peaked at 10.11%BW later at 75°. After the peaks, both shifted toward inferior shear near end-range, with rTSR reaching a greater negative value at 120° (− 15.90%BW) than aTSR (− 8.27%BW). The compressive and resultant GHJCF magnitudes decreased by 26.5% and 33.4% in rTSR across the entire motion range, respectively.

### Glenohumeral joint instability ratio

**Fig. 6:**
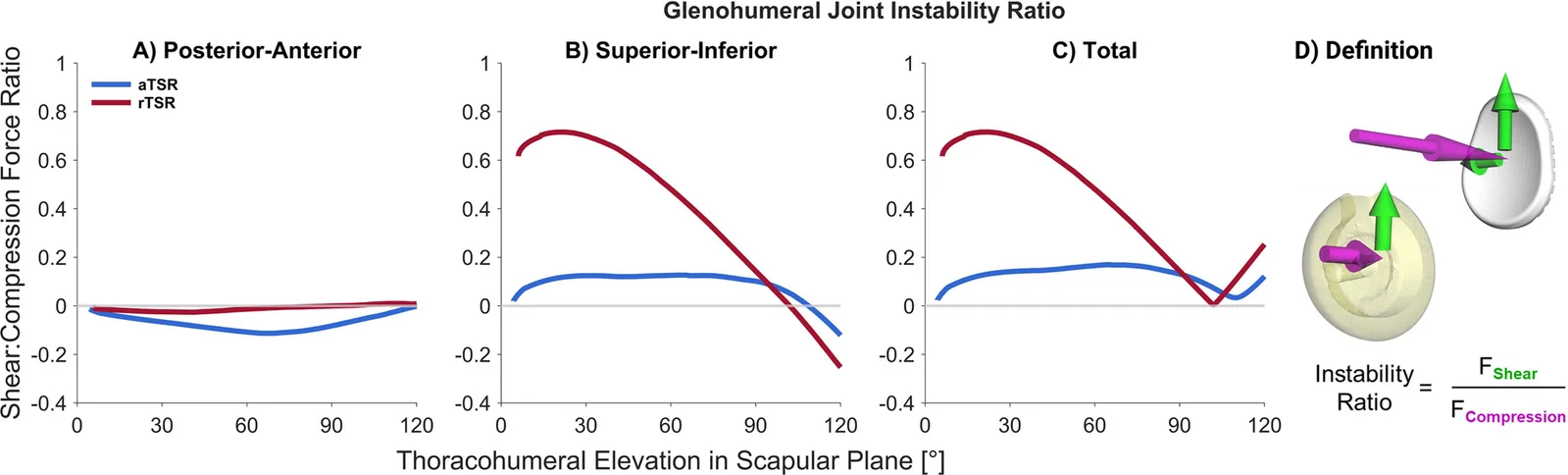
Glenohumeral joint instability ratio during arm elevation in the scapular plane for anatomic (aTSR) and reverse total shoulder replacement (rTSR). Instability ratio, defined as the shear-to-compression joint contact force ratio (GHJCF_Shear_/GHJCF_Compression_), is shown relative to the thoracohumeral elevation. Joint forces are expressed in the glenoid and glenosphere coordinate systems. **A** posterior-anterior directional instability ratio, with negative values representing posterior instability, **B** superior-inferior directional instability ratio, with positive values representing superior instability, **C** total instability ratio calculated from the resultant shear-force magnitude divided by the compression-force magnitude, and **D** schematic definition of the instability ratio

In aTSR, the instability ratio in each direction and the total ratio remained below 0.2 across the entire elevation range (Fig. 6). In the posterior-anterior direction, it rose gradually, peaked near 70° at a 0.11 ratio, then shifted gradually toward the zero line. In the superior-inferior direction, it rose rapidly to reach a peak near 0.1 at 15°, and maintained the value up to 90°. In rTSR, the total instability ratio was consistently greater than in aTSR, starting at 0.61 and peaking at 0.71. During the late-elevation phase, the total ratio decreased markedly, reached a minimum near zero, and then increased again toward the end of elevation.

The mean total instability ratio (Fig. 6C) was 69.2% lower for aTSR with 0.12 ± 0.05 than for rTSR with 0.39 ± 0.24. In the superior-inferior direction, the absolute ratio magnitude for rTSR was higher than for aTSR across almost the entire range (Fig. 6B). The instability ratio in the posterior-anterior direction for aTSR was higher than that of rTSR, which showed a very low instability ratio in this direction. However, in this direction, the mean absolute magnitude remained below 0.05 for both designs.

### Glenohumeral joint contact mechanics

**Fig. 7:**
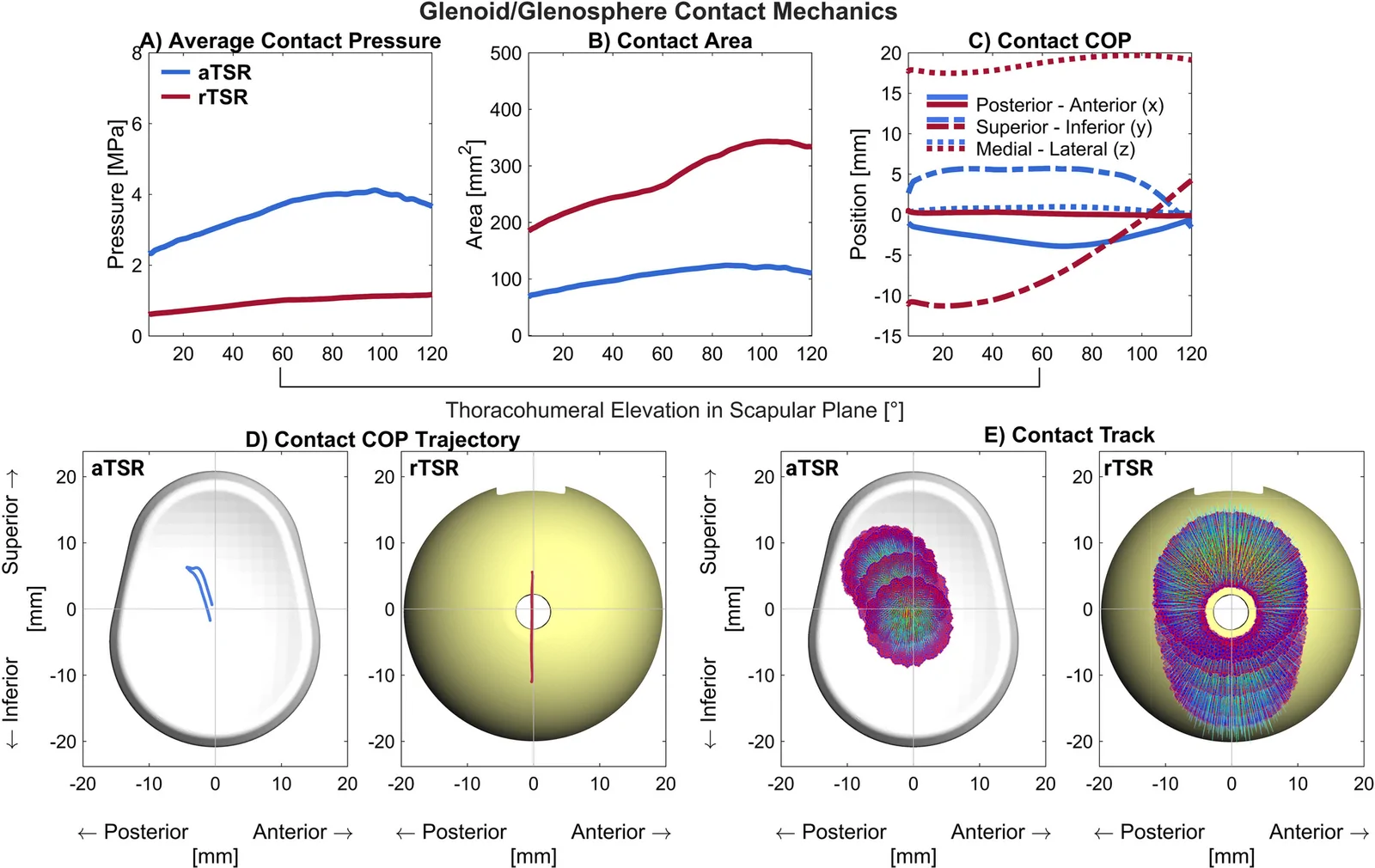
Glenohumeral joint contact mechanics during arm elevation in the scapular plane for anatomic (aTSR) and reverse total shoulder replacement (rTSR). Predictions of the RoSSiL simulation framework for **A** average contact pressure, **B** contact area, **C** center of pressure (COP) in posterior-anterior, superior-inferior, and medial–lateral directions applied to the glenoid component for aTSR and the glenosphere for rTSR. **D** displacement of the projected COP on the glenoid surface for aTSR and the glenosphere surface for rTSR calculated by the RoSSiL simulation framework. **E** footprint of the humeral head and humeral inlay on the glenoid and glenosphere. The origins in panels D and E show the coordinate systems of the glenoid component in the aTSR and the glenosphere in the rTSR

Average contact pressure was considerably higher on the glenoid component in aTSR (Fig. 7A), increasing to a peak of 4.12 MPa at 97°, whereas rTSR remained low throughout the elevation between 0.6 MPa and a peak of 1.17 MPa at the end of the motion.

The contact area was substantially larger on the glenosphere in rTSR (Fig. 7B), reaching a peak of 343.25 mm^2^ at 102°, compared with 124.12 mm^2^ at 86° on the glenoid component in aTSR.The most pronounced change in COP occurred in the superior-inferior component (Fig. 7C), where rTSR shifted from an inferior COP position of − 10.80 mm superiorly, while the COP in aTSR remained superior over most of the motion. Maximum COP displacement occurred in the superior-inferior direction for both designs, with 7.32 mm in the aTSR and 15.48 mm in the rTSR. The posterior-anterior COP position remained almost centered in rTSR, while in aTSR it remained posterior throughout the entire motion (Fig. 7C, D), with a maximum posterior shift of − 3.90 mm near 67°. The medial–lateral COP position remained comparatively stable within each design, with a range of displacement of 0.86 mm in aTSR and 2.20 mm in rTSR. However, this component showed a clear offset of around 18 mm between designs. The footprint, or the contact track for both designs, is shown in Fig. 7E. This illustrates the continuously increasing contact area in rTSR, as already quantified in Fig. 7B, and further represents the superior-inferior track of the humeral inlay on the glenosphere. This figure also shows the footprint of the humeral head on the glenoid in aTSR, dominant in the superior-posterior direction.

### Glenohumeral joint translations

**Fig. 8:**
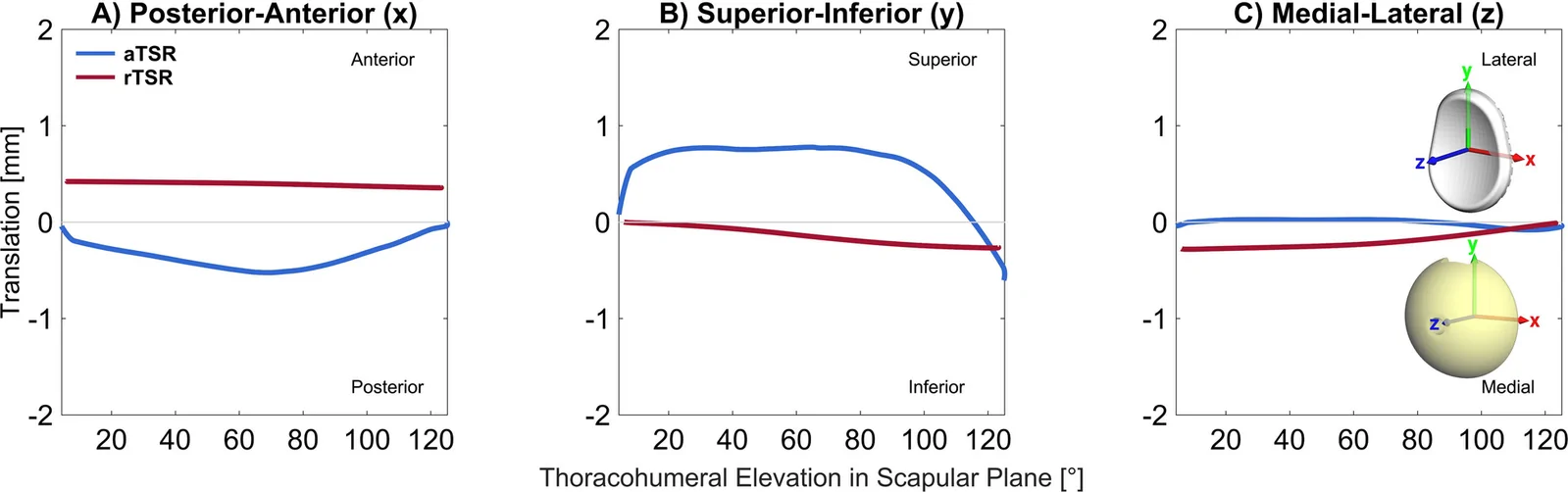
Glenohumeral joint translations during arm elevation in the scapular plane for anatomic (aTSR) and reverse total shoulder replacement (rTSR**).** Translational motion of the center of the best-fit sphere of the humeral head/inlay relative to the center of the best-fit sphere on the glenoid/glenosphere is shown. Translations are reported in **A** posterior-anterior (x), **B** superior-inferior (y), and **C** medial–lateral (z) directions for each implant design, and expressed in the glenoid and glenosphere coordinate systems as shown in panel C

The GH translations also exhibited distinct direction-dependent patterns between designs (Fig. 8). For aTSR, the largest translation magnitude was in the superior-inferior direction, and translation in the medial–lateral direction was negligible. For rTSR, however, the joint translated equally in superior-inferior and medial–lateral directions, and the posterior-anterior translation was negligible.

In the posterior-anterior direction (Fig. 8A), aTSR remained posterior across the entire elevation range, first translating posteriorly up to 70° for 0.48 mm, and then translating back to the center for 0.45 mm. However, rTSR remained anterior throughout the motion, with negligible posterior translation of 0.05 mm. In the superior-inferior direction (Fig. 8B), aTSR first translated superiorly for approximately 0.77 mm up to 25°, remained near this superior position to around 70°, and translated 0.97 mm inferiorly at the end of the motion range. In contrast, rTSR showed a consistent pattern of progressive inferior translation of 0.26 mm. Medial–lateral translations were negligible for aTSR (Fig. 8C), with a maximum of 0.1 mm medial translation at the end of the motion range. The humeral component center in rTSR started in a medial position of − 0.28 mm and moved laterally as elevation progressed.

## Discussion

The rTSR represents a larger and growing proportion of TSR [1, 44]. Despite its success rate, important design trade-offs and complications of this non-anatomic implant concept remain debated [45], and prior biomechanical studies have largely focused on isolated rTSR variables rather than providing a comprehensive quantitative comparison with aTSR. The reverse design, used in the present study, altered GH joint mechanics by shifting its COR medially and inferiorly relative to the aTSR design. This shift was expected to produce substantial changes in GH biomechanics. Therefore, an improved understanding of the biomechanical aspects of this mechanical change in rTSR compared with aTSR was investigated in the present study. To that end, using a previously validated musculoskeletal multibody simulation framework (RoSSiL) [32], we quantified biomechanical differences between the aTSR and rTSR under identical cuff conditions in a controlled comparative biomechanical study. Accordingly, all model features, including the rotator cuff condition, were kept constant, and the isolated effect of implant design was investigated. This controlled comparison was intended to represent the intact-cuff condition in selected patients, e.g., the primary GH osteoarthritis context, and was not designed to reproduce the altered muscle recruitment and joint loading associated with cuff-deficient rTSR indications. Although a direct comparison with clinical results is challenging, our study made several predictions that generally align with the limited data in the literature. We observed for the investigated designs that, compared with aTSR, rTSR: (1) shifts superior-inferior GH joint loading (Fig. 3H), (2) decreases the compressive GHJCF throughout the motion (Fig. 3I), (3) alters the middle deltoid and rotator cuff force patterns and magnitudes (Fig. 3B, D − F), (4) relies more on the deltoid muscles (Fig. 5), (5) alters articular contact footprints, and the migration of the COP (Fig. 7), and (6) varies the translation patterns with overall decreased magnitudes as a semi-constrained joint (Fig. 8).

### Muscle forces

Changing from aTSR to rTSR altered the deltoid and rotator-cuff force patterns (Fig. 3A − F), which suggests that the reverse design redistributed the mechanical requirements for arm elevation in the scapular plane toward lower middle-deltoid force [2] and less reliance on the rotator cuff muscles, while still engaging cuff stabilizers at high elevation [13]. This aligns with the established biomechanical concept and prior computational and experimental studies that a reverse design improves elevation capacity by altering deltoid leverage through COR relocation [2, 13, 16, 18, 21].

The medialization and inferiorisation of the GH joint COR in rTSR relative to aTSR increased the mechanical advantage of the deltoid for elevation and balancing the arm weight [3]. This COR medialization of 27 mm for the rTSR in the present study falls well within the reported range of 20–30 mm in the literature, which compares it to the native shoulder [3].

The force reduction due to this COR medialization in rTSR was only pronounced in the middle deltoid, which aligns with its role as the primary abductor during arm elevation in the scapular plane. Nevertheless, the middle and posterior deltoids remained the predominant muscle force generators during arm elevation in both designs (Fig. 3B, C).

The predicted 26.3% decrease in middle deltoid peak force in the present study is consistent with a 20% reduction simulated with a finite-element analysis by Terrier et al. [16]. Thus, rTSR required 73.7% of the peak middle-deltoid force predicted for aTSR to perform the same arm elevation, confirming the potential mechanical advantage of reverse design. Prior computational studies on Grammont-type reverse designs by Kontaxis and Johnson similarly report increased deltoid function during elevation as a direct consequence of the reverse geometry [21]. Experimental measurements of muscle and joint loading in human specimens by Ackland et al. [18], although conducted under deficient-cuff conditions, they also reported reduced deltoid force requirements after rTSR during elevation.

However, the reduction in middle deltoid force should not be generalized to all reverse designs. Prior cadaveric and computational studies [46, 47] have shown that glenosphere lateralization, as well as onlay or distalised configurations, can increase deltoid loading and subsequently acromial stress, providing a plausible biomechanical link to the acromial and scapular-spine stress fractures observed clinically after rTSR.

The overall reduction in rotator cuff force magnitudes in rTSR is consistent with the concept that reverse design achieves stability and motion with less reliance on rotator cuff force couples than anatomic design [2]. This reduction is supported by previous simulations [13, 19]. However, maintaining late activations in infraspinatus and supraspinatus in rTSR (Fig. 3D, F) suggests that even for an intact-cuff condition, rTSR may still recruit cuffs to maintain joint control at higher elevation, rather than eliminating their contribution, as also observed by Ackland et al. [13]. In contrast to aTSR, in which compressive GHJCF decreases after its peak around 90° (Fig. 3I), this late activation pattern in infraspinatus and supraspinatus may explain the maintained peak compressive force in rTSR.

The role of the subscapularis muscle in rTSR has remained debated, particularly because its contribution appears to vary with implant design [48]. While some studies suggest preserving it for improved postoperative stability or range of motion, others report little or no clinically meaningful benefit [48, 49]. The predicted constant and considerable decrease in the force generated by the subscapularis muscle in rTSR (Fig. 3E), in line with previous computational studies [13, 19, 28], may indicate that, by using a reverse design, the subscapularis contributes less to stabilizing the humerus than in aTSR. This interpretation is also consistent with the semi-constrained nature of rTSR, in which joint stability is achieved less through rotator-cuff force couples and more through implant conformity and deltoid-driven mechanics [2].

Regarding changes in supraspinatus muscle length, prior studies suggest that this is strongly design- and position-dependent in rTSR [50, 51]. Whereas some reverse designs and positioning have been reported to lengthen the supraspinatus substantially, the present study found a consistently shorter supraspinatus length with a medialized reverse design than in aTSR, by more than 6% (Fig. 4). Mechanically, this suggests that leaving the supraspinatus intact in rTSR may be lengthwise feasible, but not functionally equivalent to aTSR. In addition, excessive muscle stretching may impair force generation through suboptimal sarcomere overlap [50]. Postoperative supraspinatus function in intact-cuff rTSR likely depends not only on tendon preservation but also on how the selected implant geometry and position alter muscle path and length. Péan et al. showed that GH joint mechanics in rTSR are sensitive to rotator cuff integrity [20]. Therefore, the present muscle-force predictions apply specifically to the modeled intact-cuff condition and should not be extrapolated, without additional analyses, to cuff-tear arthropathy, partial or massive cuff deficiency, or revision rTSR.

### Deltoid-to-cuff ratio (DCR)

In aTSR, DCR remained close to unity in early elevation and crossed into deltoid-dominant loading only after mid-range around 58° (Fig. 5). This can be associated with increased force in the anterior and posterior deltoid muscles (Figs. 3A, C). This pattern confirms a transition from cuff-dominated loading in early elevation to deltoid-dominated loading in mid-to-late elevation in aTSR, consistent with the concept that anatomic design depends on the dynamic balance between deltoid action and cuff force couples [2, 52]. Conversely, in rTSR, a DCR of near two or higher indicates persistent large deltoid-dominant loading even under the intact-cuff assumption in the present study. This suggests a deltoid-driven solution by the reverse design for a full elevation range, confirming the key role of the deltoid in the proper function of this implant. The late DCR convergence and crossover, beyond which the ratio for aTSR exceeded that of rTSR, indicate that the two designs can adopt different muscle force balance strategies at high elevation, even when generating identical motion. In the present results, this behavior coincided with a late reduction in supraspinatus force in aTSR (Fig. 3D), while in rTSR, a non-zero supraspinatus contribution was maintained to the end range.

### Glenohumeral joint contact forces

The varied magnitudes and patterns of GHJCF (Fig. 3G − J) indicate that the two designs not only scaled the magnitude of joint loading but also redistributed implant loading direction throughout the motion. This is clinically relevant because the balance between shear and compression is closely related to joint stability [42]. The higher GHJCF in aTSR, which increases contact pressure, may elevate the risk of implant wear, particularly in the posterior region [17, 53]. In contrast, the lower GHJCF predicted for rTSR reduces mechanical loading on the glenosphere and thereby decreases the risk of glenoid loosening [3]. Notably, the compressive GHJCF component remained lower in rTSR even under the intact-cuff assumption in the present study (Fig. 3I). This may suggest that the decreased compressive GHJCF in rTSR is not only due to rotator cuff deficiency but also to the glenosphere geometry of the reverse design.

Moreover, rTSR transitioned earlier from superior to inferior shear compared to aTSR (Fig. 3H). This may indicate that the elevation phase at which the joint transitions from predominantly superiorly directed to inferiorly directed shear differed between designs. Such timing effects have also been reported to be sensitive to implant component geometry and motion in prior experimental and computational studies [18, 21].

These varied GHJCF patterns are consistent with prior reports for rTSR. They reported increased superior shear [13, 21] and reduced compression [13, 16, 21] compared to aTSR and native joint under comparable functional motions. Kontaxis and Johnson reported an average reduction in total GHJCF of 31.6% when supraspinatus, infraspinatus, and subscapularis muscles were inactive [21], whereas Masjedi and Johnson reported a 25% reduction for a full rotator cuff tear [54]. Terrier et al. and Ackland et al. reported average reductions of 30% and 32.3%, respectively, under supraspinatus-deficient conditions [13, 16]. Differences between studies should be interpreted cautiously because loading direction and magnitude depend on rotator cuff integrity, studied motion, and the muscle recruitment solution, which vary across experiments and computational modeling.

### Glenohumeral joint instability ratio

The instability ratio provided a force-based biomechanical indicator of dynamic GH joint stability, describing the balance between shear and compressive GH joint loading [42]. In the present study, with intact rotator cuffs, rTSR showed a higher total instability ratio than aTSR (0.39 ± 0.24 vs 0.12 ± 0.05) over most of the motion range (Fig. 6), consistent with findings of Curran et al. [55]. Although instability and dislocation remain important causes of revision after rTSR [56], the present force ratio alone cannot quantify this clinical risk.

Previous experimental studies defined this ratio as a measure of intrinsic joint stability, quantified as the peak translational shear force required to cause the implant subluxation or dislocation, divided by the simultaneously applied compressive force to press the implant components together [57–60]. Reported experimental ratios range from approximately 0.4 to 1.0 for aTSR [58, 59], and from 1.0 to 2.5 for rTSR [57]. These values represent design-specific resistance to subluxation or dislocation and should therefore be considered experimental reference ranges rather than universal instability thresholds. The dynamic instability ratios calculated in the present study remained consistently below these reported ranges for both designs (Fig. 6). However, this should be interpreted with caution, as the experimental ratios are dependent on the applied compression force magnitude [58, 59], the arm position and elevation phase [57], shear force direction [60], implant geometry and conformity [58], components positioning [57] and specific testing protocols.

The higher instability ratio in rTSR should not be interpreted as a direct equivalent of lower overall joint stability than in aTSR, as the ratio thresholds are different across aTSR and rTSR. In aTSR, stability depends more strongly on rotator cuff-provided concavity-compression and dynamic force balancing [2]. Therefore, a lower shear-to-compression ratio maps more directly to better dynamic centering and stability in aTSR. In contrast, rTSR benefits from an additional geometric source of stability through its more conforming, semi-constrained articulation [2, 61], which is not fully captured by such a simple force balance ratio. Thus, in rTSR, a higher instability ratio may indicate a less favorable loading balance, but this is partly compensated by implant conformity and constraint. The instability ratio should therefore be best interpreted as a force-based indicator for dynamic loading conditions rather than as a direct measure of clinical dislocation or instability risk across fundamentally different implant concepts, aTSR and rTSR.

Shoulder stability appeared to be also elevation-dependent. Goetti et al. [2] stated that the arm position most prone to instability in case of rTSR is 30° abduction. That aligns well with the early-elevation superior-inferior instability peak in rTSR (Fig. 6B). In the reverse design, the instability ratio was primarily driven by the superior-inferior force component, whereas the posterior-anterior force component played a minor role, reinforcing that the superior-inferior shear force was the principal contributor to instability risk in rTSR. This pattern is consistent with our GHJCF prediction, which showed reduced compression and increased superior shear in rTSR (Fig. 3G − J), because a higher shear-to-compression balance implies less compressive centering relative to destabilizing shear. A relatively sharp decrease in the instability ratio at the end of motion (Fig. 6C) may be related to the increased infraspinatus, supraspinatus, and posterior deltoid muscle force in rTSR (Figs. 3C − F), which stabilized the joint.

By contrast, aTSR indicated a greater absolute magnitude of the posterior-anterior instability ratio than that of rTSR (Fig. 6A), with the negative values indicating a predominantly posterior-directed shear component according to the defined coordinate-system convention, although the absolute values remained small for both designs.

In summary, the present results suggest that rTSR exhibited a less favorable shear-to-compression force balance predominantly in the superior-inferior direction, particularly at the beginning of the investigated motion, whereas in aTSR the instability ratio pattern was more balanced across directions, with the largest ratio magnitude occurring around mid-elevation. Nevertheless, these findings are valid for the intact-cuff condition in the present study and should not be generalized to other cuff conditions, which have been shown to substantially alter joint loading balance [13, 20, 62]. In this regard, Pastor et al. [62] reported decreased anterior stability for resected rotator cuffs compared to an intact condition.

Two points are important when interpreting the instability ratios. First, the metric is a force-based biomechanical indicator and does not directly simulate subluxation or dislocations. Therefore, this ratio should be interpreted as a comparative biomechanical indicator of joint loading within the investigated motion rather than as a predictor of clinical instability. Second, the ratio does not account for geometric constraint, articular conformity, soft-tissue tension, implant positioning, impingement, or patient-specific anatomy. Third, and more importantly, because the ratio depends on shear force magnitude relative to the compression force, it is sensitive to modeling choices, especially to the coordinate system in which the calculated joint contact forces are expressed.

### Glenohumeral joint contact mechanics

The joint loading in aTSR was concentrated over a smaller contact area, whereas rTSR distributed load over an approximately three times larger, more conforming interface (Fig. 7B). This was also reflected by substantially higher mean and peak contact pressure (3.5 times) in aTSR (Fig. 7A). Such a pressure-area trade-off is consistent with a prior simulation study on reverse design, which reported much lower articular contact pressures in rTSR than in aTSR [14]. This is explained by a combination of reduced joint force (Fig. 3I, J) and considerably increased contact area (Fig. 7B) when the articular surfaces are more congruent in rTSR. In the present study, the reduction in contact pressure was less extreme than the 20-fold reduction reported by Terrier et al. [14] for a Grammont-type design. This difference probably exists because our pressure measure represents average contact pressure rather than local peaks, and because loading conditions, cuff assumptions, and implant geometry differ across studies.

The COP trajectory further emphasized that the TSR designs did not simply change the load magnitude, but also altered how and in which motion phase the load was applied to the articular surface (Figs. 7C, D). In aTSR, COP remained posterior and mainly superior rather than perfectly centered across most elevation phases, with a relatively compact trajectory on the glenoid surface (Fig. 7D). The posterior edge loading in aTSR suggests potential for component loosening. This pattern is compatible with prior aTSR simulations [15], observations on retrieved glenoid components [53], and dual-plane fluoroscopic images reporting posterosuperior shifts of the contact and wear region [63]. In rTSR, however, COP showed a pronounced superior-inferior migration, while the posterior-anterior COP remained almost centered (Fig. 7C, D). These inter-design differences suggest that in rTSR, superior-inferior COP exhibited distinct elevation-dependent migration patterns, with rTSR shifting from inferior to superior at approximately 103°, whereas aTSR remained superior through most of the motion before shifting from superior to inferior at approximately 115°. This is consistent with the direction-dependent changes in GHJCF reported in the present study (Figs. 3 G − J). Such elevation-phase COP migration may matter both mechanically and clinically because it can alter the joint moment at the fixation as the load application point shifts along the glenoid and glenosphere [64]. Also, it can change which surface regions accumulate contact loading over repeated cycles, which is a key input for wear models [15], and alter contact-driven complication pathways linked to impingement and polyethylene wear, e.g., scapular notching [65]. We also observed a temporal correspondence between COP eccentricity and the shear-to-compression ratio. When the COP was positioned farther from the joint center at the beginning of the motion for rTSR and at mid-elevation for aTSR (Fig. 7C), the instability ratio also reached its highest values in those configurations (Fig. 6C), consistent with observations of Matsen et al. [66]. This temporal association indicates that eccentric contact coincided with a less compression-dominant force balance. However, one should note that this is not directly associated with clinical instability.

The combination of a larger contact area and longer COP trajectory in rTSR is relevant when interpreting polyethylene wear mechanisms. Prior simulations comparing anatomic and reverse designs reported markedly lower contact pressures in the reverse design and higher predicted volumetric polyethylene wear because the wear area was larger; increasing the contact surface can reduce linear wear while increasing volumetric wear [14, 15]. Although wear was not modeled in the present study, the observed differences in area and COP indicate that the contact mechanics metrics derived from explicit multi-point surface contact can provide input for future wear analyses. Moreover, eccentric contact and COP migration have been linked to rocking moments at the implant-bone interface and “rocking-horse” type mechanisms in previous shoulder replacement studies [16, 27]. Therefore, future work should extend the present framework to include wear prediction, enabling evaluation of the longevity of implant materials and testing their sensitivity to implant conformity, positioning, and cuff integrity under multiple functional tasks.

### Glenohumeral joint translations

Quantifying GH translations indicated how implant-specific design characteristics permit or constrain humeral component migration during elevation (Fig. 8). This is relevant as subluxation represents excessive GH translation with partial loss of joint contact, and translation patterns therefore provide a mechanical context for instability [67]. The anatomic and reverse implant designs controlled joint positioning through different mechanical strategies, even under similarly performed elevation and identical cuff conditions. It is well known that in aTSR, arm function depends on muscle force balance and on an implant design that rotates around a moving COR within the humeral head, allowing small translations. In contrast, rTSR is often designed as a semi-constrained articulation with a relatively fixed center of rotation, which limits translations [2].

Consistent with these design concepts, predictions from the RoSSiL simulation framework for rTSR in the present study showed close to zero posterior-anterior translation (Fig. 8A), while translations during elevation occurred mainly as a progressive inferior shift (Fig. 8B) and lateral migration (Fig. 8C). In contrast, the largest translations for aTSR were observed in the superior-inferior direction and remained posterior throughout the motion (Fig. 8A). The larger medial–lateral translation in rTSR than in aTSR is driven by the radius of the glenosphere geometry, which forces the humeral head to migrate more in this direction. Overall, the negligible (below 0.5 mm) translation amplitudes predicted for rTSR may represent the conforming ball-and-socket-like articulation compared to aTSR. Together, these patterns suggest that aTSR allowed a more mobile humeral head center during arm elevation in the scapular plane, while rTSR guided the humeral inlay along a more constrained path around the glenosphere.

The predominance of superior-inferior translation in aTSR aligns with previous experiments on the native GH joint in human specimens [68], and experiments on human specimens after aTSR [69], where translation of the humeral head during elevation in the scapular plane is typically greatest in the superior-inferior direction and increases as articular joint conformity decreases [70]. Matsen et al. [71] further described that, in conforming aTSR, humeral-head translation can cause articular separation and rim loading, thereby transmitting eccentric forces to the glenoid fixation and contributing to polyethylene wear, cold flow, and other deformation and component loosening. For the studied rTSR, the limited translations compared to aTSR are consistent with the deep, conforming concavity of the humeral articular surface in the reverse implant design [72], which is intended to limit GH translation and maintain a large surface contact area throughout the elevation.

### Limitations and future work

Although the present study provides insights into the biomechanical differences between aTSR and rTSR through a controlled comparison using a musculoskeletal multibody simulation framework [32], several limitations should be considered. First, the simulations were based on a single generic upper-extremity anatomy from the Visible Human Male dataset [33] rather than on a subject-specific cohort. As a result, the reported values in the present study may depend on patient morphology, muscle strength, and glenoid anatomy. Therefore, the external validity of results beyond the modeled anatomy and their applicability to a broader population remain uncertain. Future work should therefore extend the framework to include subject-specific models derived from medical images and test the findings across a larger cohort, as recent reviews identify personalization and functional model calibration as a major requirement before musculoskeletal simulation frameworks can be generalized for clinical use [73].

Second, only a single set of aTSR and rTSR implant systems from a single manufacturer and a single implant positioning was evaluated. This limits generalizability because specific design characteristics, e.g., lateralization, neck-shaft angle, and glenoid inclination, have all been reported to influence GH joint biomechanics [14, 74]. The present findings should therefore be interpreted as design-specific rather than universal for all aTSR and rTSR designs from different manufacturers. A future direction is to perform a systematic sensitivity analysis across different implant designs [75] and implant positioning [14, 74, 76, 77] within the same modeling framework to generalize these findings.

Third, the analysis was limited to arm elevation in the scapular plane with one prescribed kinematic pattern. Prior simulations and studies with instrumented implants have shown that GH joint biomechanics are strongly task-dependent [23]. The current results should therefore be interpreted as specific for standardized arm elevation in the scapular plane, not as representative of all daily activities. Future work should incorporate postoperative motion reconstructed from fluoroscopy [28] or experimental motion capture [34] and examine a wider range of clinically relevant motions, particularly positions associated with instability, such as hand-behind-head, or reaching the lower back [78].

Fourth, passive stabilizing structures, such as the capsule and ligaments, were not explicitly implemented in the simulation framework. For the present mid-range elevation task up to 120°, this simplification is unlikely to affect the presented findings [79, 80], but it may underestimate restraint near the end range and therefore affect predictions of translation or instability at more extreme arm elevations [72]. Future studies should incorporate passive stabilizing elements in the model when evaluating end-range or dislocation-prone motions. [81]

Fifth, the rotator cuff was assumed to be intact and fully functional in both TSR designs. This assumption was intentional because the study addressed selected patients with intact-cuff conditions, e.g., primary GH osteoarthritis in whom either aTSR or rTSR may be considered, and because modeling identical rotator cuff conditions between designs enabled the evaluation of the isolated effect of implant design [7, 8]. The scope of inference is therefore restricted to this intact-cuff condition. The findings should not be extrapolated to cuff-tear arthropathy, partial or massive cuff deficiency, or revision rTSR, because rotator cuff integrity substantially influences muscle recruitment, GHJCF, and joint loading balance [20, 82]. Future work should therefore repeat the same comparative analysis under graded and muscle-specific cuff-deficiency conditions [26, 27, 83].

Finally, the predicted muscle forces depend on the selected muscle recruitment strategies. Prior simulations on rTSR have noted that assuming healthy recruitment behavior and neglecting co-contraction can influence force-sharing estimates, especially in pathological or postoperative shoulders [84]. Accordingly, the present absolute values reported for muscle force magnitudes should be interpreted cautiously, even if the overall differences between studied TSR designs are plausible and consistent with previous studies. Future work should compare alternative recruitment strategies and calibrate them, where possible, using electromyography signals [85], simulators on human specimens [86], fluoroscopic imaging [28], or instrumented implant data [23].

## Conclusion

The present study used a validated musculoskeletal multibody simulation framework with a detailed representation of the glenohumeral (GH) joint. This enabled a direct comparison and quantification of the isolated effects of mechanically different designs of anatomic (aTSR) and reverse total shoulder replacement (rTSR) on GH joint biomechanics.

The results showed that the arm with two different implant designs performed similar motion through different biomechanical strategies. Particularly, rTSR altered loading toward greater deltoid dominance, lower compressive and resultant GH contact forces, less favorable shear-to-compression joint force balance, lower contact pressure, and a larger contact area. At the same time, rTSR showed distinct center-of-pressure migration and more constrained joint translations than aTSR. In contrast, aTSR preserved a more mobile humeral head center and greater dependence on rotator cuff force couples for joint centering.

This study remained limited to one implant design for aTSR and rTSR, a single investigated motion, a single generic male anatomy, and the assumption of an intact rotator cuff. Future work should therefore generalize the findings to additional implant designs, subject-specific anatomies, alternative cuff conditions, and broader functional motions. The findings may potentially provide a clinically relevant biomechanical basis for more realistic preclinical assessment of total shoulder replacements, for implant development and for hypothesis definition in experimental or clinical follow-up studies. The significance of the present study lies in its contribution to advancing understanding of the biomechanics of the GH joint following both established TSR designs through a preclinical comparative biomechanical analysis.

## Data Availability

The data supporting the findings of this study are included in the article and its supplementary material. Further inquiries may be directed to the corresponding author.

## Abbreviations

aTSR: Anatomic total shoulder replacement
BW: Body weight
CoCrMo: Cobalt chromium molybdenum
COP: Center of pressure
COR: Center of rotation
DCR: Deltoid-to-cuff ratio
DOF: Degree of freedom
GH: Glenohumeral
GHJCF: Glenohumeral joint contact force
RoSSiL: Rostock Shoulder Software-in-Loop
rTSR: Reverse total shoulder replacement
TSR: Total shoulder replacement
VEPE^®^: Vitamin-E highly cross-linked polyethylene
